# Supplementary material for: Participant Recruitment Issues in Child and Adolescent Psychiatry Clinical Trials with a Focus on Prevention Programs: A Meta-Analytic Review of the Literature
Source: J Clin Med. 2023 Mar 16;12(6):2307. doi: 10.3390/jcm12062307 (PMC10055793; doi:10.3390/jcm12062307)
Supplement: Supplementary file 1 [file jcm-12-02307-s001.zip › Supplementary 1.pdf]

Supplementary 1 List of included studies and related articles

1. Bliznak, L., Berg, R., Häge, A., & Dittmann, R. W. (2013). High rate of non-eligibility: methodological factors impacting on recruitment for a multicentre, double-blind study of paediatric patients with major depressive disorder. *Pharmacopsychiatry*, *46*(1), 23–28.
2. Boman, U. W., Broberg, A. G., Krekmanova, L., Staberg, M., Svensson, C., & Robertson, A. (2014). An explorative analysis of the recruitment of patients to a randomised controlled trial in adolescents with dental anxiety. *Swedish Dental Journal*, *38*(1), 47–54.
3. Noble, A. M., & AAKOMA Project Adult Advisory Board. (2012). Community and treatment engagement for depressed African American youth: the AAKOMA FLOA pilot. *Journal of Clinical Psychology in Medical Settings*, *19*(1), 41–48.
4. Bröning, S., Wiedow, A., Wartberg, L., Ruths, S., Haevelmann, A., Kindermann, S.-S., Moesgen, D., Schaunig-Busch, I., Klein, M., & Thomasius, R. (2012). Targeting children of substance-using parents with the community-based group intervention TRAMPOLINE: a randomised controlled trial-design, evaluation, recruitment issues. *BMC Public Health*, *12*, 223.
    - Bröning, S., Sack, P. M., Haevelmann, A., Wartberg, L., Moesgen, D., Klein, M., & Thomasius, R. (2019). A new preventive intervention for children of substance-abusing parents: Results of a randomized controlled trial. *Child & Family Social Work*, *24*(4), 537-546.
5. Cheung, K. L., ten Klooster, P. M., Smit, C., de Vries, H., & Pieterse, M. E. (2017). The impact of non-response bias due to sampling in public health studies: A comparison of voluntary versus mandatory recruitment in a Dutch national survey on adolescent health. *BMC Public Health*, *17*(1), 276.
6. Crutzen, R., Bosma, H., Havas, J., & Feron, F. (2014). What can we learn from a failed trial: insight into non-participation in a chat-based intervention trial for adolescents with psychosocial problems. *BMC Research Notes*, *7*, 824.
7. May, D. E., Hallin, M. J., Kratochvil, C. J., Puumala, S. E., Smith, L. S., Reinecke, M. A., Silva, S. G., Weller, E. B., Vitiello, B., Breland-Noble, A., & March, J. S. (2007). Factors associated with recruitment and screening in the Treatment for Adolescents With Depression Study (TADS). *Journal of the American Academy of Child and Adolescent Psychiatry*, *46*(7), 801–810.
    - March, J. S., Silva, S., Petrycki, S., Curry, J., Wells, K., Fairbank, J., Burns, B., Domino, M., McNulty, S., Vitiello, B., & Severe, J. (2007). The Treatment for Adolescents with Depression Study (TADS): long-term effectiveness and safety outcomes. *Archives of General Psychiatry*, *64*(10), 1132–1143.
8. Oesterle, S., Epstein, M., Haggerty, K. P., & Moreno, M. A. (2018). Using Facebook to Recruit Parents to Participate in a Family Program to Prevent Teen Drug Use. *Prevention Science: The Official Journal of the Society for Prevention Research*, *19*(4), 559–569.
    - Epstein, M., Oesterle, S., & Haggerty, K. P. (2019). Effectiveness of Facebook Groups to Boost Participation in a Parenting Intervention. *Prevention Science: The Official Journal of the Society for Prevention Research*, *20*(6), 894–903.

9. Schwinn, T., Hopkins, J., Schinke, S. P., & Liu, X. (2017). Using Facebook ads with traditional paper mailings to recruit adolescent girls for a clinical trial. *Addictive Behaviors*, *65*, 207–213.

   - Schwinn, T. M., Schinke, S. P., Hopkins, J., & Thom, B. (2016). Risk and protective factors associated with adolescent girls' substance use: Data from a nationwide Facebook sample. *Substance abuse*, *37*(4), 564-570.

10. Smith, K. A., Macias, K., Bui, K., & Betz, C. L. (2015). Brief Report: Adolescents' Reasons for Participating in a Health Care Transition Intervention Study. *Journal of Pediatric Nursing*, *30*(5), e165–e171.

11. Thrul, J., Stemmler, M., Goecke, M., & Bühler, A. (2015). Are you in or out? Recruitment of adolescent smokers into a behavioral smoking cessation intervention. *Addictive Behaviors*, *45*, 150–155.

12. Wagner, K. D., Asarnow, J. R., Vitiello, B., Clarke, G., Keller, M., Emslie, G. J., Ryan, N., Porta, G., Iyengar, S., Ritz, L., Zelanzny, J., Onorato, M., & Brent, D. (2012). Out of the black box: treatment of resistant depression in adolescents and the antidepressant controversy. *Journal of Child and Adolescent Psychopharmacology*, *22*(1), 5–10.

13. Young, A. S., Seidenfeld, A. M., Healy, K. Z., Arnold, L. E., & Fristad, M. A. (2018). Predicting enrollment in two randomized controlled trials of nonpharmacologic interventions for youth with primary mood disorders. *Journal of Affective Disorders*, *235*, 368–373.

    - Fristad, M. A., Vesco, A. T., Young, A. S., Healy, K. Z., Nader, E. S., Gardner, W., ... & Arnold, L. E. (2019). Pilot randomized controlled trial of omega-3 and individual–family psychoeducational psychotherapy for children and adolescents with depression. *Journal of Clinical Child & Adolescent Psychology*, *48*(sup1), S105-S118.
    - Fristad, M. A., Young, A. S., Vesco, A. T., Nader, E. S., Healy, K. Z., Gardner, W., ... & Arnold, L. E. (2015). A randomized controlled trial of individual family psychoeducational psychotherapy and omega-3 fatty acids in youth with subsyndromal bipolar disorder. *Journal of Child and Adolescent Psychopharmacology*, *25*(10), 764-774.
    - Young, A. S., Meers, M. R., Vesco, A. T., Seidenfeld, A. M., Arnold, L. E., & Fristad, M. A. (2019). Predicting therapeutic effects of psychodiagnostic assessment among children and adolescents participating in randomized controlled trials. *Journal of Clinical Child & Adolescent Psychology*, *48*(sup1), S1-S12
