# Supplementary material for: Participant Recruitment Issues in Child and Adolescent Psychiatry Clinical Trials with a Focus on Prevention Programs: A Meta-Analytic Review of the Literature
Source: J Clin Med. 2023 Mar 16;12(6):2307. doi: 10.3390/jcm12062307 (PMC10055793; doi:10.3390/jcm12062307)
Supplement: Supplementary file 1 [file jcm-12-02307-s001.zip › Supplementary 12.pdf]

Supplementary 12 Summary table of relevant information to report regarding recruitment. The goal of the following table is to provide guidelines to later facilitate recruitment efficiency analyses within a research group or for a specific population. It can also benefit researchers later working on recruitment in clinical trials. Please see third column for detailed explanation.

**1.**	**Population**	Who are you recruiting ? Clinical population (which one), control etc. - create subgroups for the following part of the table
**2.**	**Method used for recruitment**	Reference each method you use for each population in subgroups (e.g. clinical settings, clinician referral, social media, flyers etc.)
**3.**	**Number of people reached**	Represents the number of people who see or encounter your ad for the study (could be up to millions for recruitment on social media)
**4.**	**Number of people interested**	Represents the number of people who either reach out to you or to whom you give more information about the study / the ones who might undergo inclusion/exclusion criteria verifications
**5.**	**Number of people consented**	Represents the number of people/parents/care givers who actually signed consent and enrolled in the study
**6.**	**Number of participants who finished study**	Represents the number of participants who underwent all sessions and completed the whole study (this can also be separated in different steps at which the participant is seen
**7.**	**Use of monetary incentives**	Did you use monetary incentive for the study? (yes/no) and how much (per hour for example)
**8.**	**Use of vouchers**	Did you use vouchers as incentive for the study? (yes/no) and how much (per hour for example)
**9.**	**Use of feedbacks / reports**	Did you use feedbacks or reports as incentive for the study? (yes/no)
**10.**	**Use of other kind of incentives**	Did you use other kinds of incentives for the study? (yes/no) Which were they?
**11.**	**Total recruitment cost**	Total amount spent for the recruitment including research team salary (for the recruitment process) and advertisement and/or flyer costs
**12.**	**Cost per people reached**	Total recruitment cost divided by the number of people reached
**13.**	**Cost per people interested**	Total recruitment cost divided by the number of people interested
**14.**	**Cost per people consented**	Total recruitment cost divided by the number of people who consented
**15.**	**Cost per participant who finished study**	Total recruitment cost divided by the number of participants who finished study
**16.**	**Time spent on recruitment**	How long did the recruitment period last in weeks? (total recruitment period)
**17.**	**Recruitment period extended**	Was the recruitment period extended (e.g. not enough participants) ? (yes/no) and reason
**18.**	**If yes, of how much**	Of how many weeks was recruitment extended?
**19.**	**People consented after extension**	How many people consented to the study after the extended recruitment period?
