# Supplementary material for: Participant Recruitment Issues in Child and Adolescent Psychiatry Clinical Trials with a Focus on Prevention Programs: A Meta-Analytic Review of the Literature
Source: J Clin Med. 2023 Mar 16;12(6):2307. doi: 10.3390/jcm12062307 (PMC10055793; doi:10.3390/jcm12062307)
Supplement: Supplementary file 1 [file jcm-12-02307-s001.zip › Supplementary 13.pdf]

Supplementary 13. Quality assessment for the studies included.

Author (year)	Allocation sequence random?	Allocation sequence concealed?	Absence of baseline differences between groups?	Recruitment methods described?	Recruitment methods efficiency described?	Recruitment duration reported	Recruitment monetary incentives specified	Paritcipating population described?	Pariticipating rate described?	Drop out rate described?	Detailed description of baseline sample	Study duration specified?	Reponse rates at follow up reported?	Quality score %
Bliznak et al. (2013)	yes	NA	NA	yes	yes	yes	no	yes	yes	yes	NA	no	yes	62%
Boman et al. (2014)	yes	NA	NA	yes	yes	yes	no	yes	yes	yes	NA	yes	yes	69%
Breland-Noble et al. (2012)	yes	yes	no	yes	yes	no	no	yes	yes	yes	yes	yes	yes	77%
Bröning et al. (2012)	yes	NA	NA	yes	no	yes	no	yes	yes	yes	NA	yes	yes	62%
Cheung et al. (2017)	no	yes	no	yes	yes	no	no	yes	yes	no	yes	yes	no	54%
Crutzen et al. (2014)	no	NA	NA	yes	yes	yes	no	yes	yes	yes	yes	no	yes	62%
May et al. (2007)	no	yes	no	yes	yes	yes	no	yes	yes	yes	yes	yes	yes	77%
Oesterle et al. (2018)	no	NA	NA	yes	yes	yes	yes	yes	yes	yes	no	yes	yes	69%
Schwinn et al. (2017)	no	NA	NA	yes	yes	yes	no	yes	yes	yes	yes	yes	yes	69%
Smith et al. (2015)	yes	NA	no	yes	yes	no	yes	yes	no	yes	NA	yes	yes	62%
Thrul et al. (2015)	yes	yes	NA	yes	yes	yes	yes	yes	yes	yes	yes	yes	yes	92%
Wagner et al. (2012)	yes	yes	no	yes	no	no	no	yes	no	yes	no	yes	yes	54%
Young et al. (2018)	yes	yes	NA	yes	no	yes	yes	yes	yes	yes	yes	yes	yes	85%
