# Supplementary material for: Participant Recruitment Issues in Child and Adolescent Psychiatry Clinical Trials with a Focus on Prevention Programs: A Meta-Analytic Review of the Literature
Source: J Clin Med. 2023 Mar 16;12(6):2307. doi: 10.3390/jcm12062307 (PMC10055793; doi:10.3390/jcm12062307)
Supplement: Supplementary file 1 [file jcm-12-02307-s001.zip › Supplementary 2.pdf]

Supplementary 2 recruitment strategies used in the included studies to improve recruitment for children and adolescents in mental health

Author (year)	Community setting		Clinical setting		Social network				N° of strategies used
	Word of mouth / Schools	Community[a]	Research clinic	Clinician referral	Remote methods[b]	Flyers, newspapers	Social media[c]	Media[d]	
Bliznak et al. (2013)			Yes						1
Boman et al. (2014)				Yes					1
Breland-Noble et al. (2012)	Yes	Yes						Yes	3
Bröning et al. (2012)			Yes			Yes		Yes	3
Cheung et al. (2017)	Yes				Yes				2
Crutzen et al. (2014)			Yes						1
May et al. (2007)	Yes		Yes			Yes			3
Oesterle et al. (2018)	Yes						Yes		2
Schwinn et al. (2017)							Yes		1
Smith et al. (2015)					Yes				1
Thrul et al. (2015)	Yes								1
Wagner et al. (2012)				Yes					2
Young et al. (2018)	Yes			Yes		Yes			3
**N° of studies**	6	1	4	3	2	3	2	2	
**Total per category**	7		7		9				

Clinical methods encompass all that happen within clinics. Remote methods encompass all where researchers reach out to participants without meeting them. In person methods happen within a community in the participant's daily life. Paid methods are the ones where the study advertisement was costly. [a]based organizations or community members [b]phone, email, post ; [c]Facebook, Twitter, Youtube, Google ; [d]webpage, TV

and radio
