# Supplementary material for: Participant Recruitment Issues in Child and Adolescent Psychiatry Clinical Trials with a Focus on Prevention Programs: A Meta-Analytic Review of the Literature
Source: J Clin Med. 2023 Mar 16;12(6):2307. doi: 10.3390/jcm12062307 (PMC10055793; doi:10.3390/jcm12062307)
Supplement: Supplementary file 1 [file jcm-12-02307-s001.zip › Supplementary 3.pdf]

Supplementary 3 Recruitment promotion and outcomes for the studies included to improve recruitment strategies for children and adolescents in mental health

Author (year)	Recruitment methods	Recruitment period (in weeks)	Patient involvement	People reached n	Reached rate (participant per month)	Recruitment consented (%)	Finished study (%)
Bliznak et al. (2013)	Clinical	56	only inclusion/exclusion procedure	85	6.1	1 (1.18)	1 (100)
Boman et al. (2014)	Clinical	138	1 dentist appointment	104	3.0	55 (52.88)	55 (100)
Crutzen et al. (2014)	Clinical	32	when needed	290	36.3	12 (4.14)	0 (0)
Wagner et al. (2012)	Clinical	NA	24 weeks	NA	NA	334 (NA)	185 (55.39)
Bröning et al. (2012)	Clinical + Social	182	9 sessions	302	6.6	218 (72.19)	178 (81.65)
Breland-Noble et al. (2012)	Community + Social	NA	120 minutes	23	NA	17 (73.91)	16 (94.12)
Cheung et al. (2017)	Community + Social	NA	45 minutes	NA	NA	11312 (NA)	NA
Oesterle et al. (2018)	Community + Social	12	24 minutes	235	78.3	103 (43.83)	84 (81.55)
Schwinn et al. (2017)	Social	24	15 minutes	1873	312.2	797 (42.55)	788 (98.87)
Smith et al. (2015)	Social	NA	10 minutes	68	NA	26 (38.24)	25 (96.15)
Thrul et al. (2015)	Community	52	465 minutes	1054	81.1	273 (25.90)	272 (99.63)
May et al. (2007)	Community + Clinical + Social	156	12 weeks	2804	73.8	439 (15.66)	178 (40.55)
Young et al. (2018)	Community + Clinical + Social	136	12 weeks	119	3.5	95 (79.83)	73 (76.84)

NA, Not Available
