# Supplementary material for: Participant Recruitment Issues in Child and Adolescent Psychiatry Clinical Trials with a Focus on Prevention Programs: A Meta-Analytic Review of the Literature
Source: J Clin Med. 2023 Mar 16;12(6):2307. doi: 10.3390/jcm12062307 (PMC10055793; doi:10.3390/jcm12062307)

Supplementary 4 recruitment rate of available studies (n=11) separated by reached population. The recruitment rate represents the number of people who signed the informed consent form out of those who were reached out to.

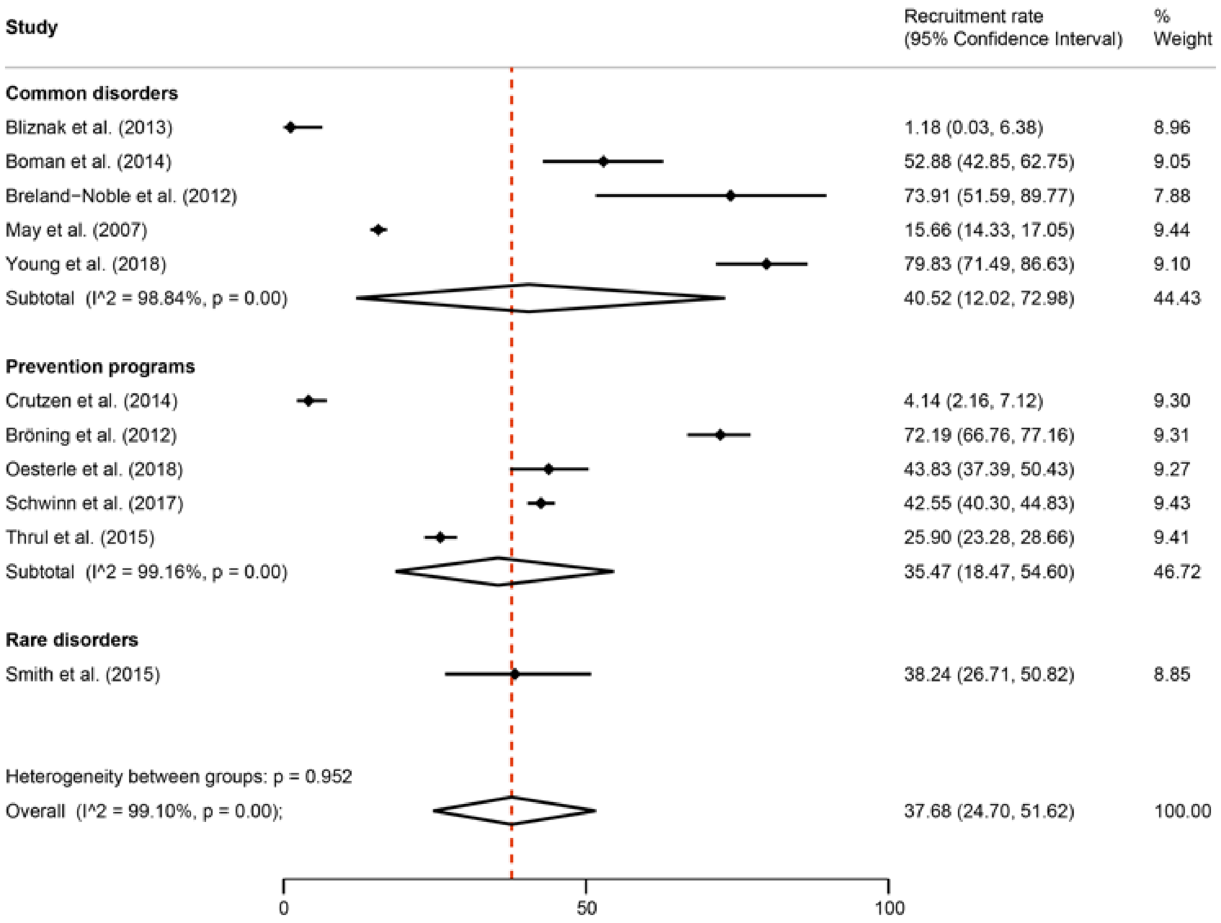

Supplement: Supplementary file 1 [file jcm-12-02307-s001.zip › Supplementary 4.pdf]
