# Supplementary material for: Participant Recruitment Issues in Child and Adolescent Psychiatry Clinical Trials with a Focus on Prevention Programs: A Meta-Analytic Review of the Literature
Source: J Clin Med. 2023 Mar 16;12(6):2307. doi: 10.3390/jcm12062307 (PMC10055793; doi:10.3390/jcm12062307)

Supplementary 6. Recruitment rate of available studies (n=11) separated by the number of recruitment methods used. The recruitment rate represents the number of people who signed the informed consent form out of those who were reached out to.

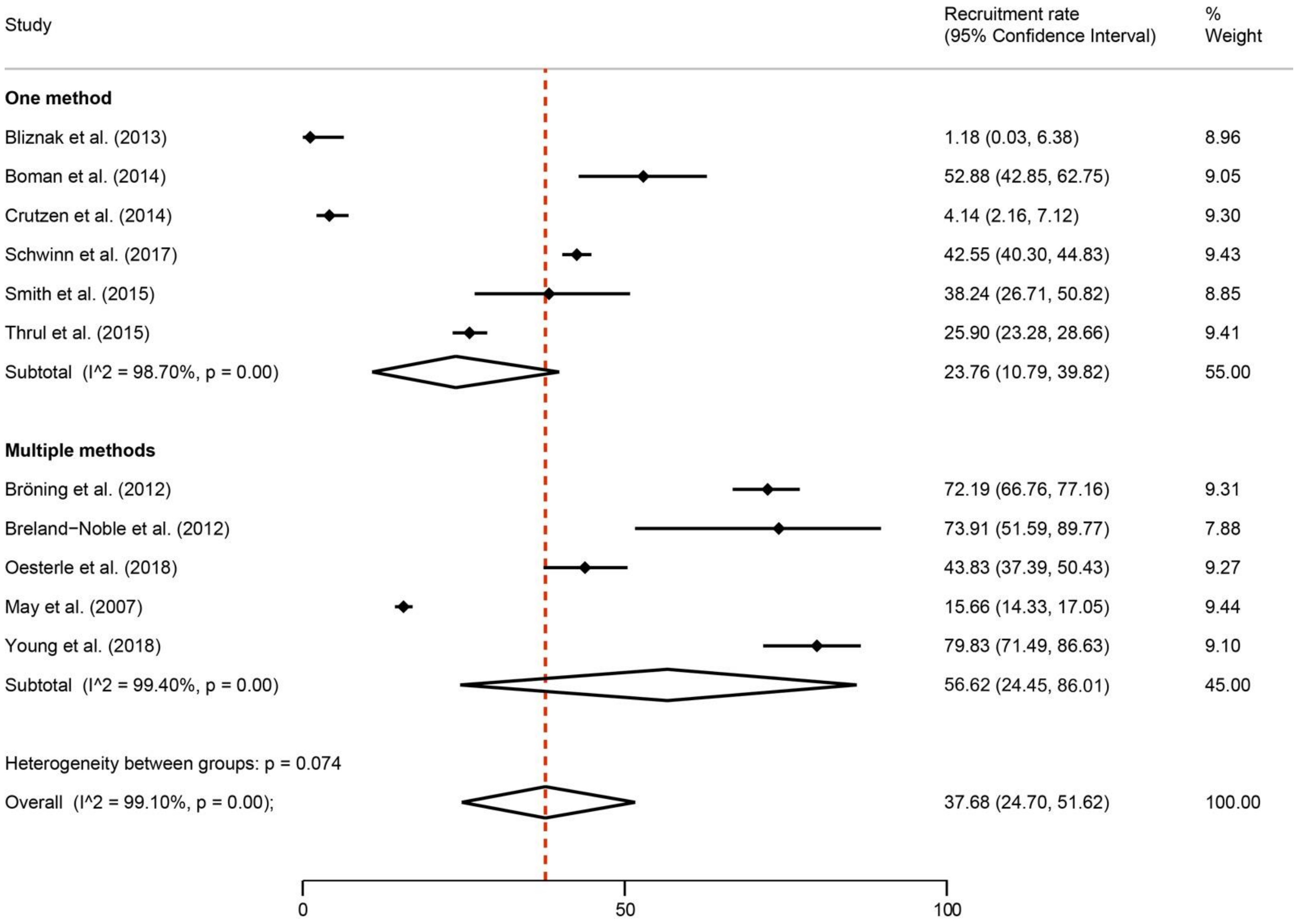

Supplement: Supplementary file 1 [file jcm-12-02307-s001.zip › Supplementary 6.pdf]
