# Supplementary material for: Participant Recruitment Issues in Child and Adolescent Psychiatry Clinical Trials with a Focus on Prevention Programs: A Meta-Analytic Review of the Literature
Source: J Clin Med. 2023 Mar 16;12(6):2307. doi: 10.3390/jcm12062307 (PMC10055793; doi:10.3390/jcm12062307)

Supplementary 9 completion rate of available studies (n=12) separated by the use of monetary incentives. The completion rate represents the number of participants who finished the study with all timepoints available, over the number of recruited.

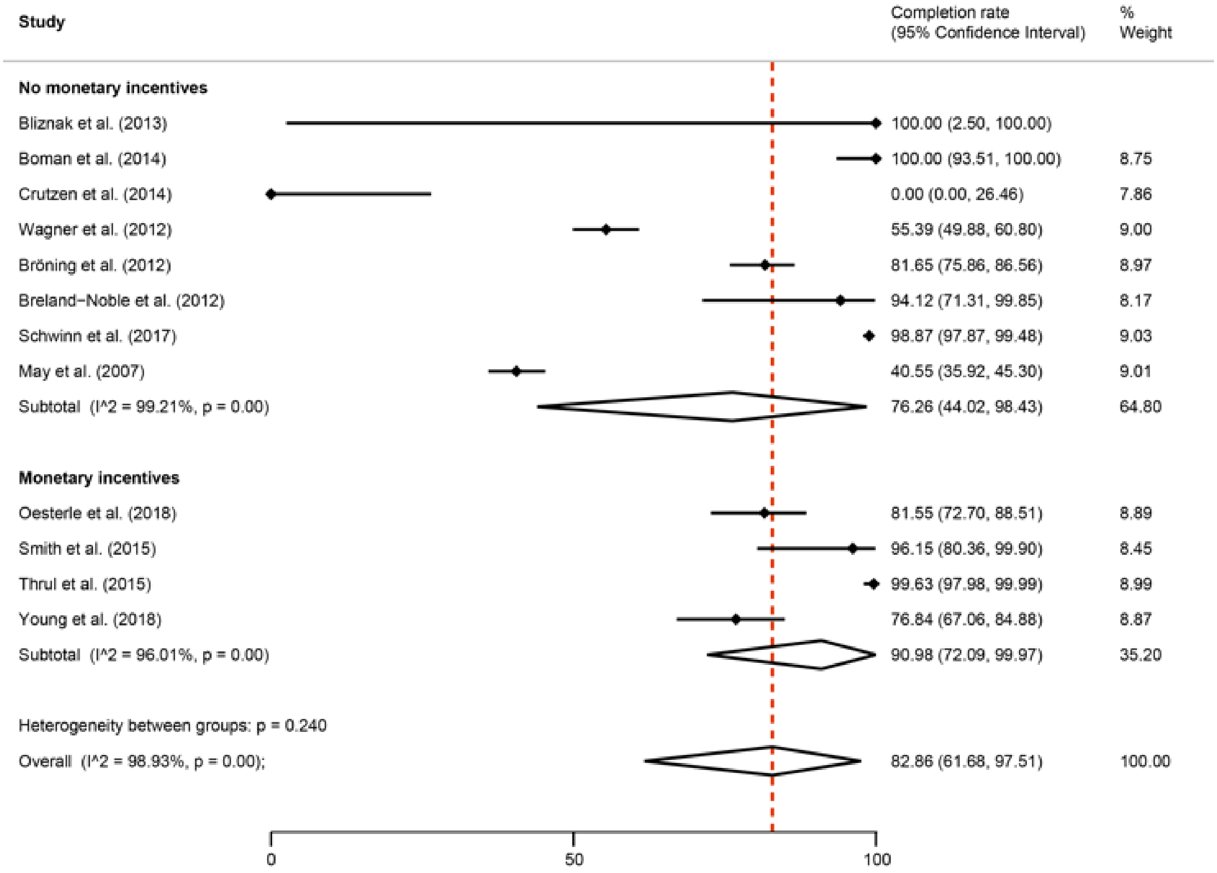

Supplement: Supplementary file 1 [file jcm-12-02307-s001.zip › Supplementary 9.pdf]
